# Research on the effect of TiO_2_ nanotubes coated by gallium nitrate on *Staphylococcus aureus*‐*Escherichia coli* biofilm formation

**DOI:** 10.1002/jcla.23417

**Published:** 2020-09-08

**Authors:** Junjie Dong, Bing Wang, Bingquan Xiang, Jin Yang, Zhiqiang Gong, Zhihua Wang, Yunchao Huang, Lingqiang Chen

**Affiliations:** ^1^ Department of Orthopedics The First Affiliated Hospital of Kunming Medical University Kunming China; ^2^ Department of Thoracic Surgery The Third Affiliated Hospital of Kunming Medical University Tumor Hospital of Yunnan Province Kunming China; ^3^ The First Affiliated Hospital of Kunming Medical University Kunming China

**Keywords:** biofilm, *Escherichia coli*, gallium nitrate, *Staphylococcus aureus*, TiO_2_ nanotubes

## Abstract

**Background:**

In clinical practice, the cases with bacterial infection caused by titanium implants and bacterial biofilm formation on the surface of titanium materials implanted into human body can often be observed. Thus, this study aimed to demonstrate whether the mixed biofilm of *Staphylococcus aureus*/*Escherichia coli* can be formed on the surface of titanium material through in vitro experiments and its formation rules.

**Methods:**

The titanium plates were put into the well containing *S aureus* or/and *E coli*. Bacterial adhesion and biofilm formation were analyzed by crystal violet, XTT method, confocal laser scanning microscopy, and scanning electron microscopy.

**Results:**

The results of bacterial adhesion in each group at 6‐72 hours showed that the number of bacterial adhesion in each group was increased with the extension of time and reached to the highest level at 72 hours. Moreover, the biofilm structure in the *S aureus*‐*E coli* group was significantly more complex than that of the simple *S aureus* group or *E coli* group, and the number of bacteria was also significantly increased in the *S aureus*‐*E coli* group.

**Conclusion:**

Those data provide a laboratory basis for the prevention and treatment of mixed infection of subsequent biological materials.

## 1 INTRODUCTION

In 1951, the application of titanium in orthopedics was first reported that titanium, as an inert metal, was the ideal material for fracture fixation.^1^ Later, titanium was used in oral surgery because of its good biocompatibility. However, due to its low biological strength, poor wear resistance was limited to use in weight‐bearing bone. With the development of materials science, titanium alloy came into being, overcoming the shortcomings of pure titanium. Because of its high strength and good biocompatibility, it is widely used in fracture fixation. In the early stage, Ti‐6Al‐4V and i‐3AL‐2.5V were applied in the field of orthopedics, but due to their toxic elements, which might lead to toxic reactions, they were gradually replaced by alloy materials such as α + β and β alloy materials.^2^ Titanium and its alloy materials have stronger biomechanical properties and histocompatibility than stainless steel and other materials in the past. At present, titanium and its alloy materials are the most commonly used materials in orthopedics and stomatology. It is of great clinical significance to study the infection caused by its implantation from the most commonly used materials. However, due to the fact that titanium alloy materials are composed of a variety of elements and there are many interference and confounding factors, it is difficult to carry out the subsequent nano‐modification for titanium alloy materials. Therefore, pure titanium metal was selected as the material for the in vitro study in this experiment, so as to provide ideas and laboratory basis for the subsequent study of titanium alloy and nickel‐cobalt alloy.

With the abuse of antibiotics, the emergence of drug‐resistant strains and the high incidence of some immunodeficiency diseases, mixed infection has become a difficult problem in clinical work, especially for the diseases in intensive care unit such as infections caused by various catheter implantation, orthopedic diabetic foot, and chronic osteomyelitis caused by internal fixation implantation. Houshian et al reviewed and studied 418 patients with hand surgical infection, with a mixed infection rate of 11.7%.^3^ A large number of studies at home and abroad have shown that the mixed bacterial biofilm formed on the surface of the material after the mixed bacterial infection has more complex morphology and biological characteristics.^4^ However, the related research on the mixed biofilm of *Staphylococcus aureus* and *Escherichia coli* on the surface of titanium metal material has not been reported at home and abroad. Therefore, in this study, titanium plates were mixed with *S aureus*‐*E coli* to observe whether mixed biofilms could be formed on the surface of titanium and the formation rules and characteristics. In addition, a control study was conducted with a single bacterial biofilm to explore the causes of complex, severe, and refractory mixed infection, hoping to provide a laboratory basis for the prevention and treatment of mixed infection of subsequent biological materials.

## 2 MATERIALS AND METHODS

### 2.1 Preparation of titanium plate materials

The pure titanium plates with 5 × 5 mm in size are selected, polished with sandpaper in order of 800 mesh, 1000 mesh, 1200 mesh, and 2000 mesh, washed with deionized water for 15 minutes, and immersed in HF + HNO_3_ + H_2_O = 1:1:8 solution for 15 seconds to carry out chemical polishing. After that, deionized water was added to stop the reaction and washed for 15 minutes. Then, titanium plates were taken out and put into steam autoclave sterilizer, with a pressure of 103.4 kPa (1.05 kg/cm^2^) and a temperature of 121.3°C, for 30 minutes, and then taken out and dried in the drying oven for 30 minutes, and placed for standby.

### 2.2 Bacterial culture

*Staphylococcus aureus* and *E coli* were purchased from American Type Culture Collection (ATCC). The standard strains of *S aureus* (ATCC 25923) and *E coli* (ATCC 25922) were inoculated to MH agar plate and cultured for 24 hours at 37°C. Subsequently, 12 mL tryptic soy broth (TSB) culture medium was put into the test tube. The inoculation ring was used to select a single colony on the bacteria plate of each group (repeat for three times) and gently vibrate the liquid level junction in the test tube. The bacteria in each group were inoculated into the tube containing 12 mL TSB medium, and the tube was placed in a constant humidity oscillator at 37°C and 150 r/min for 16‐18 hours. After the growth of bacterial cells to the logarithmic growth stage, the concentration of bacterial solution in each group was adjusted to 1 × 10^7^ CFU/mL for later use with ultraviolet spectrophotometer and TSB medium. The mixed bacterial solution from mixed group was prepared according to 1:1, such as 2 mL mixed bacterial solution (1 mL *S aureus* bacterial solution + 1 mL *E coli* bacterial solution).

### 2.3 Experimental grouping

The experiment was divided into four groups: TSB‐treated control group (Group A), *E coli*‐treated group (Group B), *S aureus*‐treated group (Group C), and *E coli* and *S aureus* mixture‐treated group (Group D). 4 sterile 24‐well culture plates were taken, and three wells were selected in each plate and added 2 mL 1 × 10^7^ CFU/mL prepared bacterial solution (TSB for control group, TSB containing *S aureus*, TSB containing *E coli*, and TSB containing *S aureus* + *E coli* for experimental groups), the prepared sterilized titanium plates with the size of 5 mm × 5 mm were put into the well containing bacterial solution, and titanium plates were immersed into the bacterial solution with sterilizing tweezers and cultured in a 37°C incubator. After cultured for 6, 12, 24, 48, and 72 hours, titanium plates were taken out for bacterial biofilm determination and analysis.

### 2.4 Semiquantitative detection of bacterial adhesion and biofilm formation by crystal violet (CV)

After cultured for 6, 12, 24, 48, and 72 hours, 100 μL bacterial solution was drawn from the corresponding wells, respectively. The absorbance was measured at 620 nm with a multifunctional microplate reader and the data was recorded for standby. The corresponding titanium plates were taken out, transferred to the new 24 well plate, and added 2.5 mL PBS to rinse twice to remove the floating bacteria on the metal plate. After gently washing the bacteria and discarding PBS, the titanium plates were transferred to the absorbent paper, put into the clean 24‐well plate after air drying, added 400 μL 2% CV dye solution to dye each well, and incubated at 37°C for 30 minutes. Then, CV dye solution was sucked and discarded, and 2.5 mL PBS was added to rinse for 3 times. The titanium plate was transferred to the absorbent paper, dried at room temperature, and transferred to a new 24 well plate. After adding 400 μL DMSO to decolorize for 15 minutes, 100 μL decolorizing solution was taken from each well to 96 well plate. The optical density (OD) value of 570 nm absorbance was measured by microplate reader. The statistical chart of bacterial adhesion at different time points and groups was made with the culture time as *X* axis and 570 nm absorbance as *Y* axis. The average OD of the TSB‐treated control well was subtracted from the measured value of the well, and the measured value of the OD was corrected. Formula biofilm formation (BF) = (AB − CW)/G was used to evaluate the ability of biofilm formation. AB is the OD value of bacteria at the 570 nm, CW is the OD value of TSB‐treated control group at 570 nm, and G is the OD value of bacterial solution at the 620 nm.^5^, ^6^ BF ≥ 1.1 represents strong biofilm, BF between 0.7 and 1.09 represents mature biofilm, BF between 0.35 and 0.69 represents weak biofilm, and BF < 0.35 represents no biofilm formation.

### 2.5 Detection for the dynamic of bacterial biofilm formation by XTT method

After 6, 12, 24, 48, and 72 hours of culture, the titanium plates at the corresponding time points were taken out, transferred to the new 24 well plate, and gently washed twice with 4°C sterile 2.5 mL PBS to remove the floating bacteria on the metal plate. After discarding PBS, the titanium plates were dried naturally and then put into the 24‐well plate. Each well was added with 300 μL TSB culture medium and 60 μL XTT solution, and incubated in 37°C at dark for 2 hours. After the incubation, 120 μL medium was taken out from each well to measure the cell activity of the bacteria.

### 2.6 Observation of bacterial biofilm formation thickness and capacity by confocal laser scanning microscopy (proportion of live and dead bacteria)

The titanium plates were taken out for 12, 24, 48, and 72 hours after culture and gently washed with 4°C sterile 2.5 mL PBS twice to remove the floating bacteria on the metal plate. Then, 400 μL fluorescent dye solution (SYTO9 and PI dyes for staining living bacteria and dead bacteria, respectively) was added and dyed at room temperature for 15 minutes. After that, the titanium plates were taken out and put into 2.5 mL physiological saline for light rinsing. After absorbing the excess fluorescent dye, the titanium plates were put on the slide to fluorescently imaged under the confocal laser scanning microscopy (CLSM). Observation conditions: The argon ion laser is used as the light source. The green fluorescence excitation wavelength is 488 nm, the emission wavelength is 519 nm, the red fluorescence excitation wavelength is 559 nm, and the emission wavelength is 567 nm. Observation index: bacterial biofilm thickness, three‐dimensional reconstruction image, the percentage of live bacteria on the biofilm surface at each time point was calculated according to the area occupied by green fluorescence of live bacteria and red fluorescence of dead bacteria. Each piece of metal shall be observed in 3 visual fields, and the number of layers shall be recorded by layer scan. The above operations shall be carried out in dark conditions.

### 2.7 Ultrastructural observation of bacterial biofilm by scanning electron microscope (SEM)

The titanium plates were taken out for 6, 12, 24, 48, and 72 hours, washed with 2.5 mL PBS, and fixed in refrigerator at 4°C for 24 hours. Then, the plates were washed with PBS solution for 3 times and fixed with 1% starving acid solution at 4°C for 2 hours. After that, the titanium plates were dehydrated with ethanol gradient for 20 minutes, replaced with isoamyl acetate for 20 minutes, and frozen at −20°C following permeating with tert‐butyl alcohol at 40°C for 2 hours. The formation of biofilm on the surface of the specimen was observed under SEM.

### 2.8 Statistical analysis

SPSS19 statistical software was used for analysis. The data are expressed as mean ± standard deviation. The pairwise comparison of multiple groups was performed by ANOVA. *P* values < .05 were considered statistically significant.

## 3 RESULTS

### 3.1 Bacterial adhesion on titanium surface

**Figure 1:**
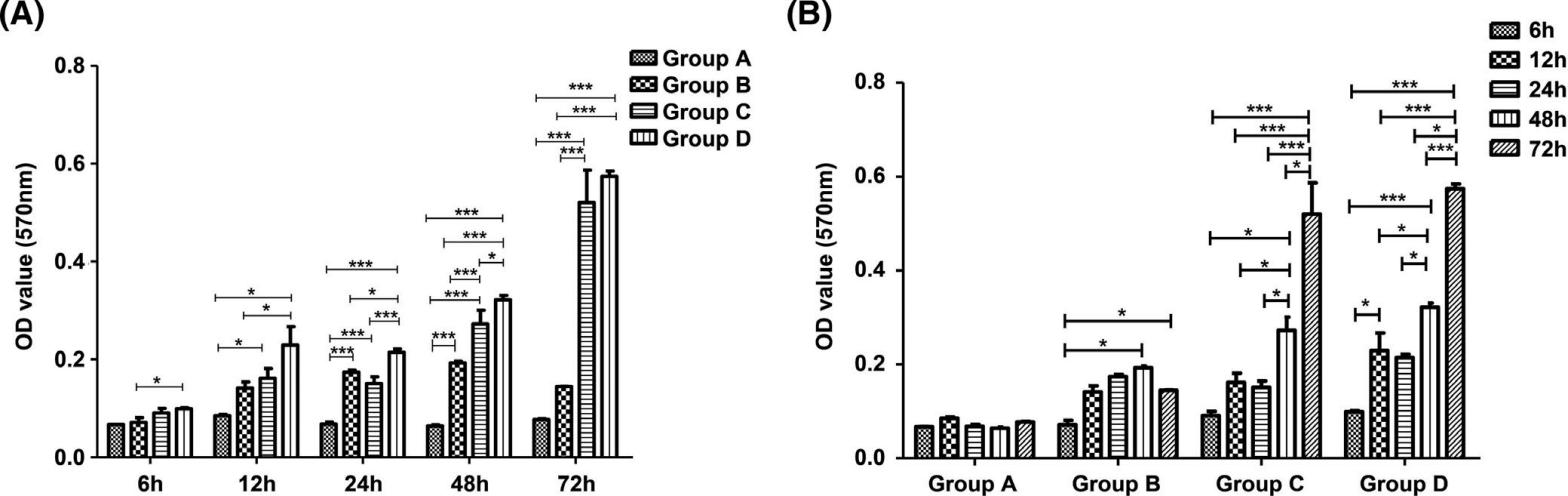
Bacterial adhesion on titanium surface in different groups by crystal violet staining. A, Results of bacterial adhesion quantitative at different time points. B, Results of bacterial adhesion quantitative of different groups at 6‐72 h. (*P < .05; **P < .01; ***P < .001)

The four groups were co‐cultured with titanium plates for 6, 12, 24, 48, and 72 hours, and semiquantitative detection of bacterial adhesion on the surface of titanium plates was conducted. The results showed that the mixed group showed the trend of the largest amount of bacterial adhesion at each time point (Figure 1A). The TSB‐treated control group, *E coli* group, *S aureus* group, and mixed group were co‐cultured with titanium tablets. The detection results of bacterial adhesion in each group at 6‐72 hours showed that the number of bacterial adhesion in each group increased with the extension of time, and the number of bacterial adhesion in each group was the highest at 72 hours incubation (Figure 1B).

### 3.2 Results of ability of bacterial biofilm formation at different time points on the surface of titanium plate

**Figure 2:**
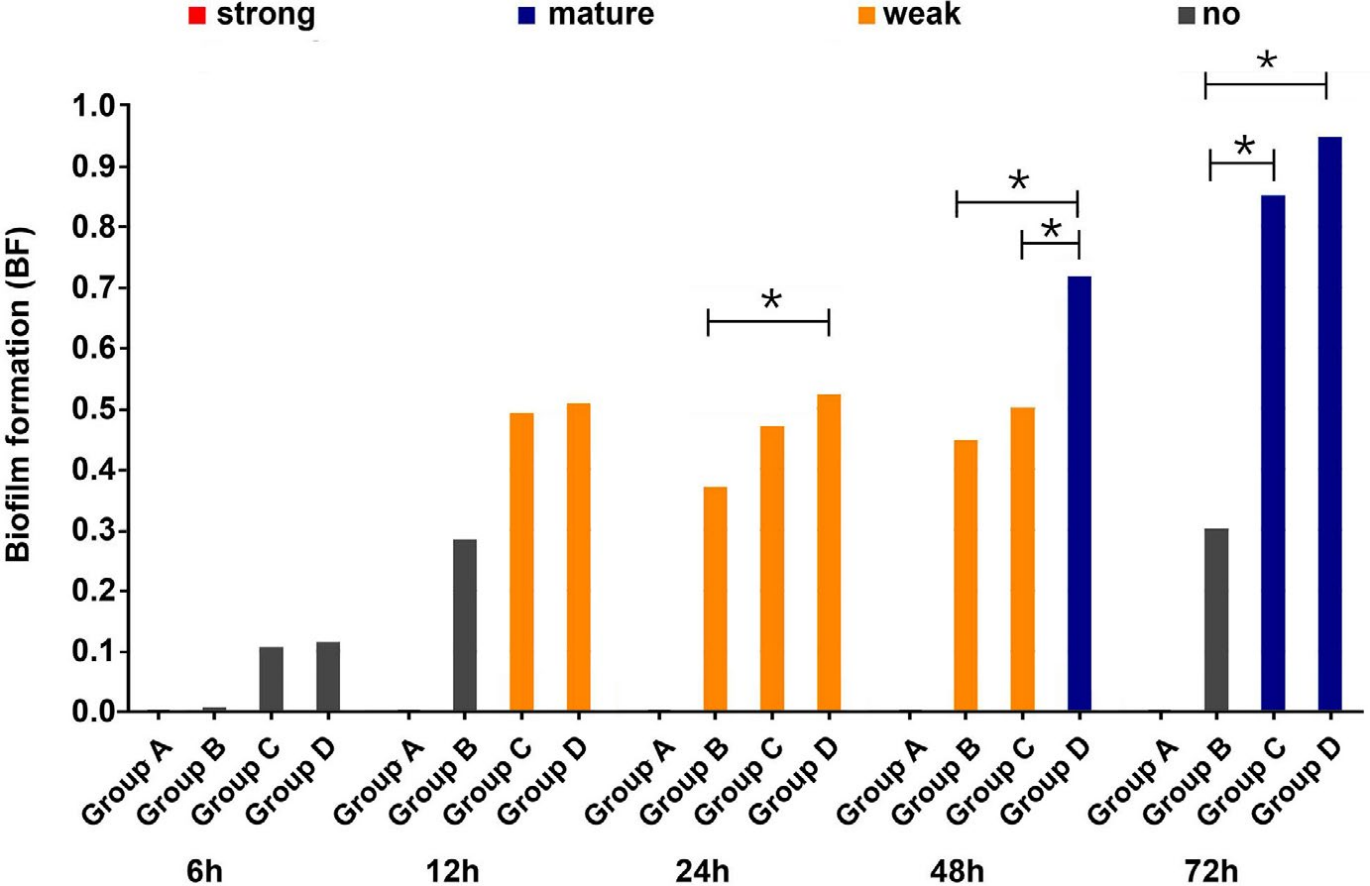
Results of ability of bacterial biofilm formation (BF) at different time points on the surface of titanium plate. BF = (bacteria OD_570 nm_ − background OD_570 nm_)/bacteria OD_620 nm_. BF value ≥ 1.1 represents strong biofilm, BF value between 0.7‐1.09 represents mature biofilm, BF value between 0.35 and 0.69 represents weak biofilm, and BF value < 0.35 represents no biofilm formation (*P < .05)

As shown in Figure 2, no bacterial biofilm was formed in *E coli* group, *S aureus* group, and mixed group at 6 hours; no bacterial biofilm was formed in *E coli* group at 12 hours, but weak bacterial biofilm was formed in *S aureus* group and mixed group at 12 hours, and there was no significant difference between the two groups (*P* > .05); weak bacterial biofilm was formed in *E coli* group and *S aureus* group at 24 hours, mature bacterial biofilm was formed in mixed group at 24 hours, and it was found that there was a statistically significant difference between the *E coli* group and the mixed group (*P* < .05), while there was no statistically significant difference in other groups (*P* > .05). At 48 hours, weak bacterial biofilm was formed in *E coli* group and *S aureus* group, and mature bacterial biofilm was formed in mixed group, which was statistically significant compared with *E coli* group and *S aureus* group (*P* < .05). At 72 hours, mature bacterial biofilm was formed in *E coli* group, *S aureus* group, and mixed group, which was statistically different between *E coli* group and *S aureus* as well as mixed group (*P* < .05), and there was no statistical significance between *S aureus* group and mixed group (*P* > .05).

### 3.3 Test results of the forming dynamic of titanium bacterial biofilm

**Figure 3:**
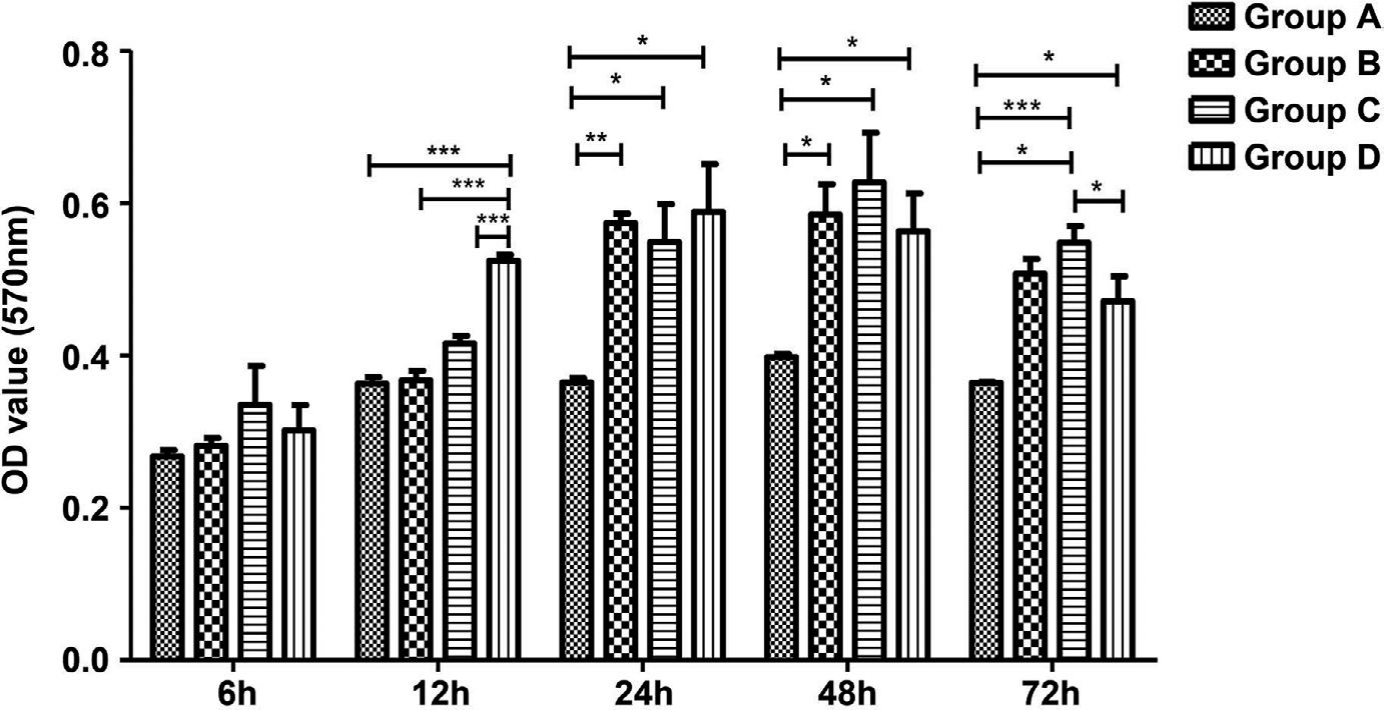
Results of the forming dynamic of titanium bacterial biofilm in each group. The bacteria activity in each group at each time point was measured by XTT cell proliferation assay (*P < .05; **P < .01; ***P < .001)

The TSB‐treated control group, *E coli* group, *S aureus* group, mixed group, and titanium plate were cultured for 6, 12, 24, 48, and 72 hours, and the dynamic detection results of biofilm formation by XTT method. The results showed that only the biofilm‐forming dynamic in the mixed group at 12 hours > *E coli* group,> *S aureus* group and > control group. There was no significant difference between mixed group and *S aureus* group as well as *E coli* group at 6, 24, 48, and 72 hours, which indicated that there was no obvious advantage in forming dynamic of biofilm by mixing *S aureus* and *E coli* (Figure 3).

### 3.4 Detection results of bacterial biofilm on titanium surface by confocal laser scanning microscope (CLSM)

**Figure 4:**
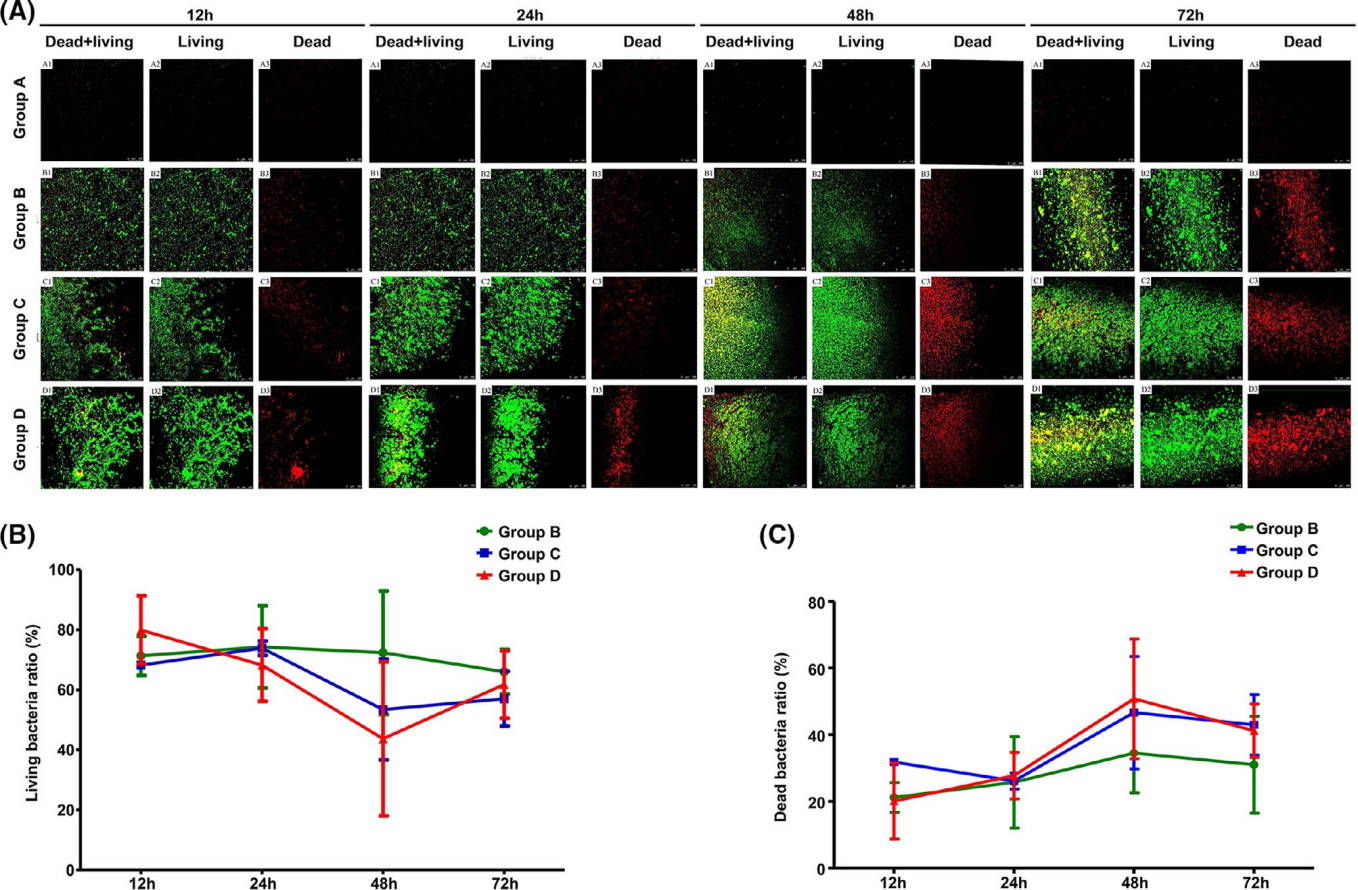
CLSM observation of bacterial biofilms of each group at each time point. The SYTO9 (green fluorescence) and PI (red fluorescence) dyes were used to stain living bacteria and dead bacteria, respectively. A, Representative CLSM images of bacterial biofilms in each group at each point. B, Living bacteria ratio of bacterial biofilms of each group at 12‐72 h. C, Dead bacteria ratio of bacterial biofilms of each group at 12‐72 h

After forming bacterial biofilm on titanium surface at different time point, SYTO9 (green fluorescence) and PI (red fluorescence) dyes were used to stain living bacteria and dead bacteria, respectively. As shown in Figure 4A, there were several punctate green and red fluorescence signals in the TSB‐treated control group, but no bacterial biofilm formation. The formation of mature bacterial biofilm was observed in the *E coli* group; a large area of green fluorescence signal was observed. Similarly, the formation of mature bacterial biofilm was observed in the *S aureus* group; a large area of green fluorescence signal with dense lamellar arrangement can be seen. The complex bacterial biofilm structure was observed in the mixed group. The mature biofilm structure was found in *E coli* group, *S aureus* group, and mixed group at 72 hours, and the mixed group was the most complex. The number of dead bacteria in each group was shown after 12, 24, and 48 hours incubation, and the mixed group had the highest number (Figure 4B,C). After 12, 24, 48, and 72 hours of culture, the proportion of living bacteria decreased and the proportion of dead bacteria increased with the prolongation of culture time in each group, among which the proportion of living bacteria in 48 hours mixed group was the least and the proportion of dead bacteria was the most (Figure 4B,C). At 12 hours, the biofilm of *S aureus*, *E coli*, and mixed groups was initially formed.

**Figure 5:**
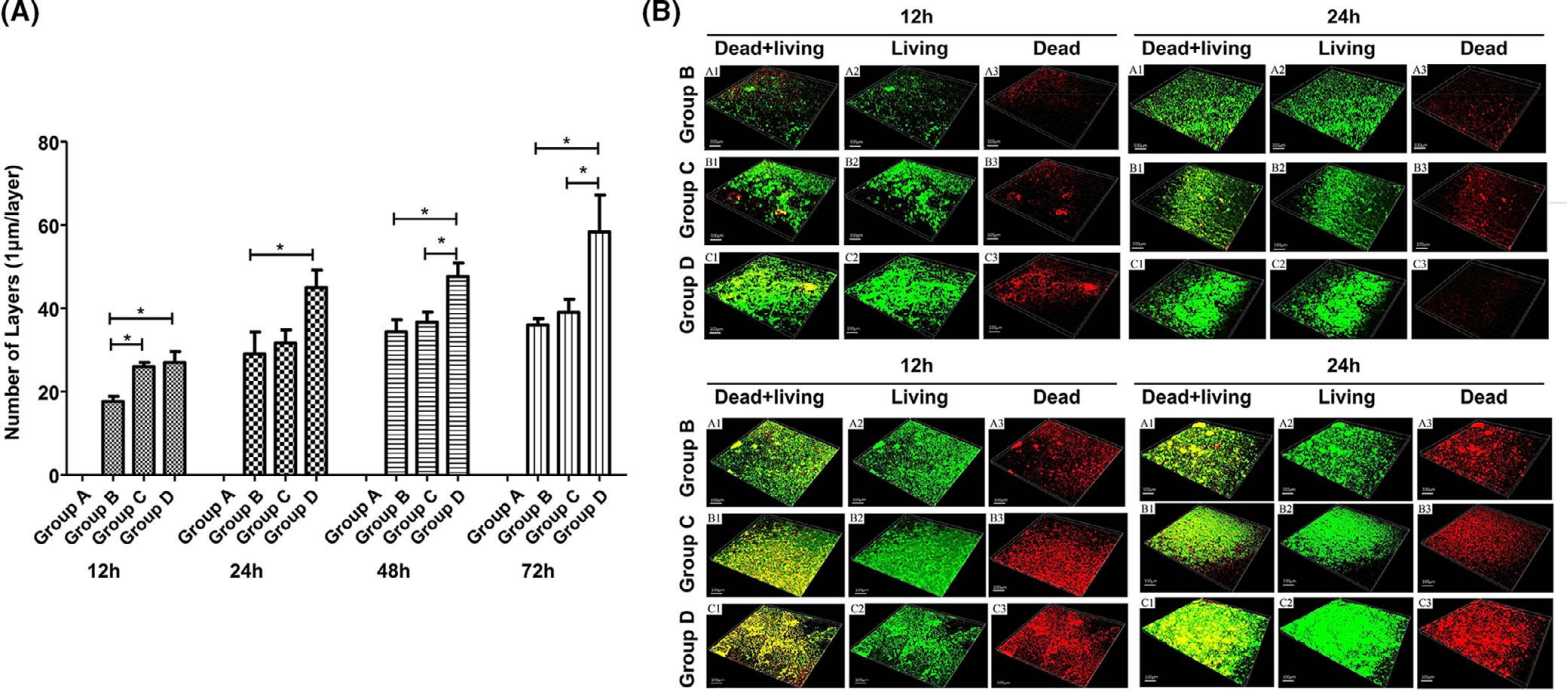
The thickness of biofilm in each group at each point. A, The thickness of biofilm in different groups at 12‐72 h. B, Representative three‐dimensional reconstruction images of bacterial biofilms in each group at each point (*P < .05)

Compared with the two groups, the thickness of mixed group was greater than that of *E coli* group, but showed no statistical differences when compared with *S aureus* group. At 24 hours, the biofilm gradually thickened, and the thickness of the mixed group was greater than that of *E coli* group, and there were no statistical differences between mixed and *S aureus* groups. At 48 hours, the thickness of biofilm increased slowly, and the thickness of the mixed group was larger than that of *S aureus* group and *E coli* group, which showed a significant difference. At 72 hours, the thickness of the biofilm was the thickest, and the thickness of the mixed group was larger than that of *S aureus* group and *E coli* group, which showed a significant difference (Figure 5A). The three‐dimensional reconstruction showed that during the co‐culture period of 12‐72 hours, the number of bacterial biofilms in the three groups of *S aureus*, *E coli*, and mixed culture increased with the prolongation of culture time, and the structural complexity also increased significantly with the prolongation of culture time, in which the amount of bacteria in the mixed group was the most and the most complex at each time point. The proportion of live bacteria in the three groups of *S aureus*, *E coli*, and mixed culture decreased and the proportion of dead bacteria increased with the prolongation of culture time (Figure 5B). Among them, the proportion of dead bacteria in the mixed group was the highest, and the proportion of dead bacteria in each group was the highest after 72 hours of culture (Figure 5B).

### 3.5 Observation results of bacterial biofilm on titanium surface by Scanning electron microscopy (SEM)

**Figure 6:**
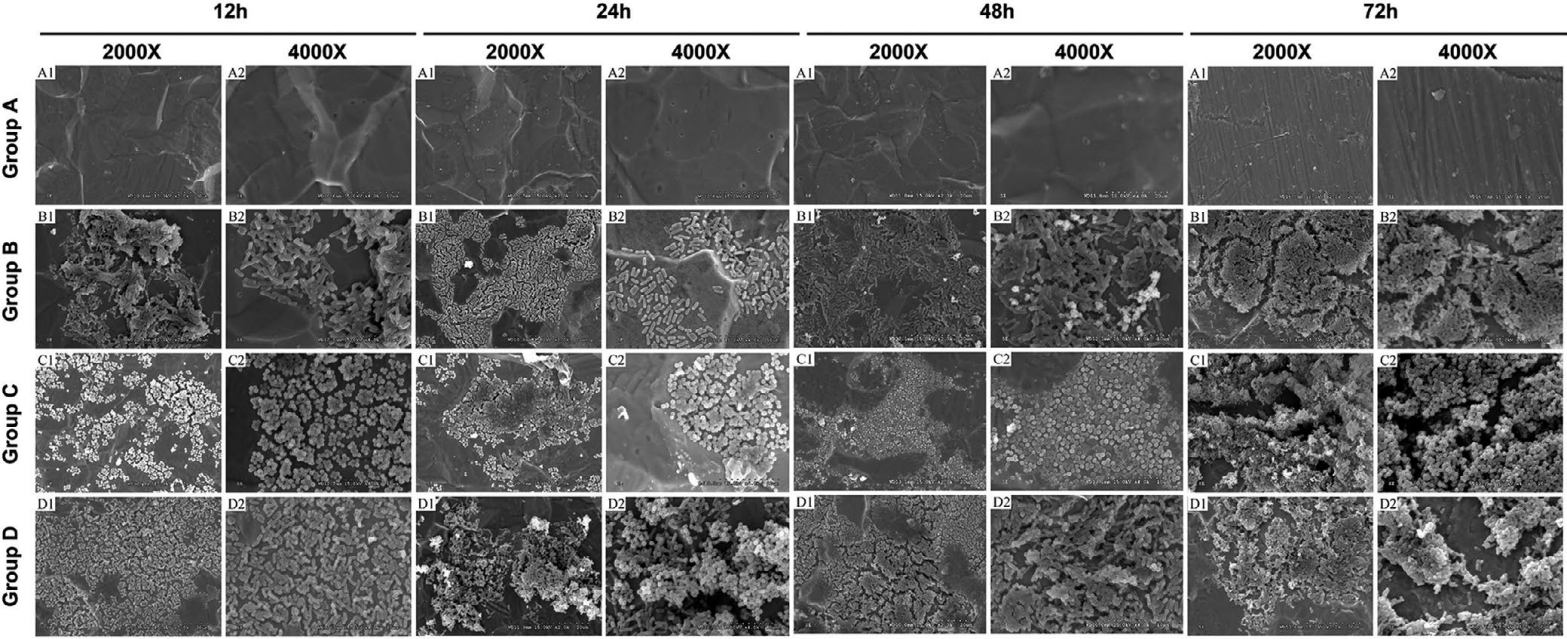
SEM observation of bacterial biofilm on titanium surface at different time points. The representative SEM images in different groups were taken at 2000× and 4000× magnifications

Mature biofilm was observed in the mixed group, *S aureus* group, and *E coli* group. In the mixed group, it can be observed that the stacked masses formed by *S aureus* and *E coli* grew together. The biofilm structure in the mixed group was significantly more complex than that of the simple *S aureus* and *E coli* group, and the number of bacteria was also significantly increased (Figure 6).

## 4 DISCUSSION

In this paper, pure titanium was used as the carrier of biomaterial, *S aureus*/*E coli* mixture was used as the source of mixed bacterial infection, and the formation of mixed biofilm was observed and analyzed by scanning electron microscopy (SEM) and confocal laser scanning microscopy (CLSM) after culture. The results showed that *S aureus*/*E coli* solution could form biofilms when co‐cultured with pure titanium plate. The results of crystal violet (CV) and XTT showed that weak mixed biofilm could be formed in 12 and 24 hours after culture, and mature mixed biofilm could be formed in 48 and 72 hours after culture. The number of mixed biofilm increased gradually with the prolongation of culture time, but the dynamic of mixed biofilm decreased with the prolongation of culture time. Through the CLSM observation, it is found that the structure of the mixed bacterial biofilm is from simple to complex, and it is formed into linear, strip, block, large and clump from scattered point like aggregation, and the thickness of the bacterial biofilm is gradually thickened. It was observed by SEM that the structure of the mixed bacterial biofilm changed from single to complex, from scattered single bacteria to agglomerated and stratified distribution. Besides, it was also observed that *E coli* was the main bacteria in the mixed biofilm of *S aureus* and *E coli*.

From the point of view of mixed infection, the study on the formation of *S aureus*/*E coli* mixed biofilm on the surface of titanium plate has certain innovation. In the past, only a few studies focused on the formation of mixed biofilm, and only limited to food, water, plants, animals, etc, and the materials studied are mostly limited to polyethylene, silica gel, glass, food, etc.^7^, ^8^ Previous studies have shown that bacterial biofilm in nature can be composed of single, double, or multiple microorganisms, and can form a single or three‐dimensional structure, and double or multiple biofilm is the main form of bacterial biofilm in nature.^9^ At present, the vast majority of studies on mixed biofilm are concentrated on mixed gram‐negative, mixed gram‐positive or mixed biofilm composed of bacteria and fungi.^10^ Barros et al take nano‐hydroxyapatite as the carrier and mix with *E coli*/*S aureus* mixed bacterial solution (1.25 × 108 cells/mL) for 24 hours to form a mixed bacterial biofilm. Compared with a single biofilm, the amount of mixed biofilm is increased, and the number of *E coli* in the mixed biofilm is more than that of *S aureus*.^11^ The research of Makovcova et al^12^ shows that the double bacterial biofilm can be formed when *S aureus* and non‐pathogenic *E coli* are mixed in culture. González et al^13^ mixed culture of *S aureus* and *Enterococcus faecium*, compared with the single culture of *S aureus*, there was no significant difference in the amount of biofilm, compared with the single culture of *E faecium*, and the amount of mixed biofilm was higher than that of *E faecium*.

It is clear that *S aureus* and *E coli* can form mixed biofilm on the surface of titanium plate, but the following problem is the relationship between the two strains in the mixed biofilm. At the same time, the formation of single bacterial biofilm and mixed biofilm was studied. The results showed that the number of *S aureus*/*E coli* mixed biofilm was more than that of single biofilm at each time point, but the forming dynamic (bacterial activity) was stronger than that of single biofilm only at 12 hours, there was no difference between single biofilm at 24 and 48 hours, and the dynamic of mixed biofilm at 72 hours was less than that of single biofilm. At the same time, the CLSM results also showed that the number of mixed biofilm increased, but the proportion of dead bacteria increased, and the activity of biofilm decreased. SEM observation showed that there were not the same number of two strains in the mixed biofilm, but mainly *E coli*. The above results indicate that after the formation of mixed organisms, their activity is inhibited and the majority of them are *E coli*. Maybe, there is a competitive relationship between *S aureus* and *E coli*, which makes the *E coli* gain the competitive advantage.

By analyzing the above results and referring to the relevant literature at home and abroad, the domestic and foreign literature reports are similar to the results of this experiment. It can be clear that in titanium materials, *S aureus* and *E coli* can grow together, and can form a more complex structure and a larger number of mixed bacterial biofilm, but there is a competitive relationship between them in the mixed biofilm. It is reported that the growth of *S aureus* in the mixed biofilm culture of *S aureus* and *E coli* is inhibited.^14^ Previous studies have shown that bacteria in nature can form cooperative (synergistic), competitive (antagonistic), or neutral relationship,^15^ and through cooperative polymerization or metabolic cooperation, it can increase the resistance to antibiotics or host immune response,^16^ while the antagonistic effect is based on the competition for nutrition and growth inhibition, which can reduce the activity of bacteria, or even inhibit or perish.^17^ Therefore, the coexistence of different bacteria in the mixed biofilm will affect the formation of bacterial biofilm. Millezi et al^18^ found a competitive relationship between *S aureus* and *E coli*, and the presence of *E coli* reduced the number of *S aureus* cells living in the biofilm. Similarly, Pompermayer and Gaylarde^14^ studied the adhesion of *S aureus* and *E coli*, and concluded that there is a competitive relationship between *E coli* and *E coli*, and *E coli* has a competitive advantage. However, the results of this experiment and Barrose's^11^ experiment show that under the effect of this competitive relationship, the mixed biofilm has more advantages than the single biofilm in both quantity and structure. The author believes that the reason lies in that this competitive effect is not mutual inhibition of extinction, but is beneficial to one side, the other side is weakened, and the products of cell disintegration for the weakened side may provide the beneficial side with the material needed for growth. The possible reasons for this competitive relationship are as follows: The author observed that in the preparation of bacterial suspension, the number of *E coli* increased significantly after 16‐18 hours of bacterial culture with the same amount of bacteria than that of *S aureus*, indicating that the time for the formation of *E coli* is shorter and the number is more, which may enable it to develop and maintain advantages in the mixed biofilm. In addition, *S aureus* in mixed biofilms may also provide some adhesion factors that *E coli* does not have, so that *E coli* can maintain dominant growth in mixed biofilms. These factors may be the reason why *E coli* keeps the advantage in the mixed biofilm and *S aureus* is inhibited in the mixed infection. In order to verify the relationship between the two, the future experiments can separate and quantitatively analyze *S aureus* and *E coli* in the mixed bacterial biofilm, and further explore the mechanism and role of their competition.

Christensen et al^5^ first described the quantitative analysis of bacterial biofilm with CV staining in 1985, then improved its accuracy and modified it, and made the biofilm quantified in the well plate.^19^ CV is an alkaline dye that binds to negatively charged surface molecules and polysaccharides in the extracellular matrix.^20^ Because bacteria, whether living or dead, and the substrate are stained with CV, it is not suitable to evaluate the activity of biofilm.^21^ In this experiment, CV method was used to detect the total amount of bacterial biofilm, and the results showed that the total amount of mixed biofilm was larger than that of single biofilm, but it contained dead bacteria, which could not reflect the activity of the overall bacterial biofilm in real time. In order to distinguish living bacteria from dead ones, detect and quantify the metabolic activity in living bacteria, the use of various reactive dyes has become a feasible technology and method, including 5‐cyano‐2,3‐ditolyl tetrazolium chloride, and XTT.^22^, ^23^ XTT method is based on the reduction of XTT dye to water‐soluble methionine.^24^ The absorbance of bacterial supernatant is in direct proportion to the number of metabolic active microbial cells. XTT method has been widely used in quantitative analysis of cells and bacteria in planktonic culture. The results of this experiment also showed that although the amount of mixed bacterial biofilm measured by CV staining method was larger than that of single biofilm, XTT found that there was no difference between mixed biofilm and single biofilm at 24 and 48 hours, but the dynamic of mixed biofilm at 72 hours was smaller than that of single biofilm, which indicated that although the total amount of mixed bacterial biofilm was large, the number of active bacteria in mixed biofilm was reduced.

CLSM is a tool widely used in biofilm observation, because it can obtain three‐dimensional images of biofilm structure and monitor its development over time. The fluorescent dyes SYTO9 and PI are a kind of nucleic acid dye. SYTO9 makes the living bacteria emit green fluorescence, while PI makes the dead bacteria emit red fluorescence. It diffuses passively through the cell membrane and binds with the DNA of the living and dead cells.^25^ Since DNA is also an important component of extracellular matrix,^26^ this staining will provide information about the total biofilm biomass. SYTO9 was used to study the composition and morphology of biofilm by CLSM.^27^ The dye is also used for conventional quantification of bacterial and yeast biofilm biomass.^28^, ^29^ Compared with single biofilm at the same time point, the thickness of mixed biofilm is thicker, the morphology is more complex, and the proportion of dead bacteria in mixed biofilm is increased, and the number of living bacteria is reduced and the number of dead bacteria is increased with the extension of culture time. SEM can show the detailed surface morphology of microbial biofilm and its structure. The SEM images of this experiment confirmed the CLSM analysis. The morphology of the mixed biofilm of *S aureus* and *E coli* is more complex than that of a single biofilm. The biofilm is composed of rod‐like clusters and layered. The number is less, and the morphology is more complex and dense with the increase of culture time.

In conclusion, quantitative and qualitative analysis methods were used to observe the formation of *S aureus*‐*E coli* mixed biofilm, and the rules of its formation were summarized and discussed in these experiments. Compared with single bacterial biofilm, the number and shape of mixed biofilm were more complex, and changed with the change of culture time. The formation process of mixed biofilm is closely related to the interrelationship between the two bacteria. Why they form complex mixed biofilm and what molecular mechanism regulates their formation process, which will be studied in the future.

## AUTHOR CONTRIBUTION

Junjie Dong and Yunchao Huang served as guarantors of integrity of the entire study and designed the study concepts. Junjie Dong, Yunchao Huang, and Lingqiang Chen designed the study. Junjie Dong and Lingqiang Chen defined the intellectual content. Junjie Dong, Bing Wang, Bingquan Xiang, and Jin Yang involved in literature research. Junjie Dong, Bingquan Xiang, Jin Yang, and Zhiqiang Gong contributed to experimental studies. Junjie Dong, Bingquan Xiang, and Zhihua Wang contributed to data acquisition. Junjie Dong, Bingquan Xiang, and Zhihua Wang analysed the data. Jin Yang, Zhiqiang Gong, and Bingquan Xiang performed statistical analysis. Junjie Dong, Zhihua Wang, and Zhiqiang Gong prepared the article. Junjie Dong, Lingqiang Chen, and Zhiqiang Gong edited the article. Junjie Dong, Jin Yang, and Zhiqiang Gong reviewed the article.
