#  

**DOI:** 10.1002/jcla.24079

**Published:** 2021-11-19

In Volume 34, Issue 11 (2020), the Figure 4 was incorrect in the article titled “The role of *Bacillus acidophilus* in osteoporosis and its roles in proliferation and differentiation” by Chen Chen, Baokang Dong, Yuming Wang, Qiang Zhang, Bangmao Wang, Shuzhi Feng, and Yu Zhu.

Incorrect Figure 1

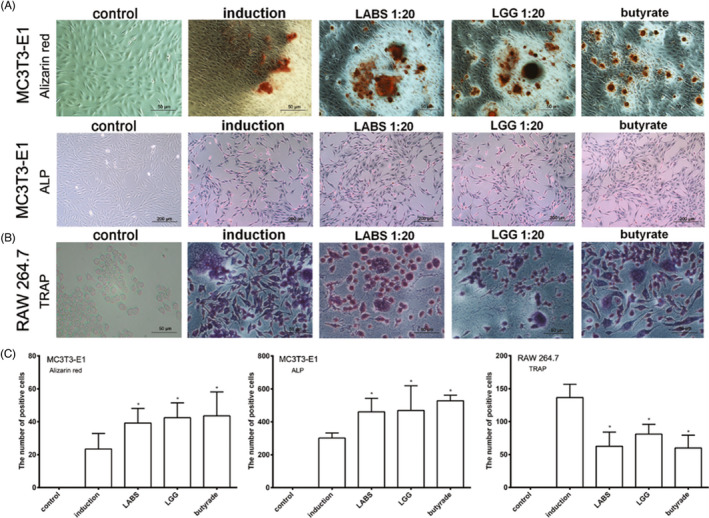

Correct version Figure 1

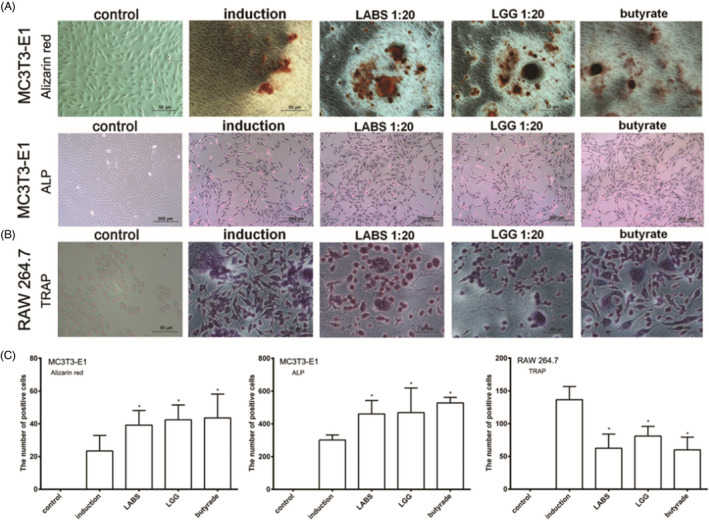

The author regrets for this error.

